# Extracellular vesicle PD-L1 in reshaping tumor immune microenvironment: biological function and potential therapy strategies

**DOI:** 10.1186/s12964-021-00816-w

**Published:** 2022-01-28

**Authors:** Jiaxing Liu, Xueqiang Peng, Shuo Yang, Xinyu Li, Mingyao Huang, Shibo Wei, Sheng Zhang, Guangpeng He, Hongyu Zheng, Qing Fan, Liang Yang, Hangyu Li

**Affiliations:** Department of General Surgery, The Fourth Affiliated Hospital, China Medical University, Shenyang, 110032 China

**Keywords:** PD-L1, Extracellular vesicles, Immune escape, Biomarker, Immunotherapy

## Abstract

Programmed cell death 1 ligand 1 (PD-L1) is the ligand for programmed death protein-1 (PD-1), is associated with immunosuppression. Signaling via PD-1/PD-L1 will transmits negative regulatory signals to T cells, inducing T-cell inhibition, reducing CD8^+^ T-cell proliferation, or promoting T-cell apoptosis, which effectively reduces the immune response and leads to large-scale tumor growth. Accordingly, many antibody preparations targeting PD-1 or PD-L1 have been designed to block the binding of these two proteins and restore T-cell proliferation and cytotoxicity of T cells. However, these drugs are ineffective in clinical practice. Recently, numerous of studies have shown that, in addition to the surface of tumor cells, PD-L1 is also found on the surface of extracellular vesicles secreted by these cells. Extracellular vesicle PD-L1 can also interact with PD-1 on the surface of T cells, leading to immunosuppression, and has been proposed as a potential mechanism underlying PD-1/PD-L1-targeted drug resistance. Therefore, it is important to explore the production, regulation and tumor immunosuppression of PD-L1 on the surface of tumor cells and extracellular vesicles, as well as the potential clinical application of extracellular vesicle PD-L1 as tumor biomarkers and therapeutic targets.

**Video Abstract**

**Supplementary Information:**

The online version contains supplementary material available at 10.1186/s12964-021-00816-w.

## Background

PD-L1 (also known as B7H1 and CD274) is a 40-kDa type 1 transmembrane protein, expressed in a variety of cells and has the greatest immunosuppressive effect when it is expressed on tumor cells [1]. Under normal conditions, the immune system reacts to foreign antigens collected in lymph nodes or the spleen and promote the proliferation and differentiation of cytotoxic T cells. When PD-L1 on tumor cells is highly expressed under the regulation of various factors, it can combine with PD-1 on the surface of T cells and transmit inhibitory signals, leading to T cell dysfunction or depletion, and then promote tumor immune escape [2, 3].

Intercellular communication is crucial under both physiological and pathological conditions. Cells mainly communicate through direct contact and the release of soluble factors, including growth factors, cytokines, and hormones. Recently, a novel method of intercellular communication involving the release of extracellular vesicles (EVs) was identified and has attracted increasing research interest [4, 5]. Many drugs targeting PD-1/PD-L1 have been developed aiming to attenuate their immunosuppressive effects; to date, however, their therapeutic benefits have been limited [6, 7], likely due to a special form of PD-L1 that is present on the surface of EVs [8, 9]. EVs comprise any type of membrane-bound vesicle that is released by cells and can be generally divided into two subgroups—exosome and microvesicle—depending on the diameter and method of formation [10].

## The physiology of EVs

### Exosome biogenesis and secretion

The diameter of exosomes ranges from 40 to 160 nm [10]. Invaginated plasma membrane buds off to form early-sorting endosomes (ESEs), which fuse with trans-Golgi network-derived vesicles that can contain cytoplasmic molecules. ESEs mature into late-sorting endosomes (LSEs) through exchanging materials, and then into multivesicular bodies (MVBs) which contain intraluminal vesicles (ILVs) formed by the inward invagination of the endosomal limiting membrane. MVB maturation occurs through at least two mechanisms, one involves endosomal sorting complex required for transport (ESCRT)-0, -I, -II, and -III and proteins associated with ESCRT-III (e.g., VPS4 and ALIX); in the other, which is independent of ESCRT, ILVs and MVBs are generated through lipids, ceramides, four transmembrane protein families, heat shock proteins, and others [11–13]. Once MVBs have been produced, they can either fuse with lysosomes or autophagic lysosomes, which leads to the degradation of MVBs; or they can fuse with the plasma membrane and be released to out of the cells as exosomes [14, 15]. Exosome secretion mainly depends on the auxiliary activity of the Rab and soluble N-ethylmaleimide-sensitive factor attachment protein receptor (SNARE) protein families [16]. The Rab protein family comprises small GTPases, several of which regulate vesicle transport and fusion through GTP/GDP cycling [17, 18]. For instance, the regulators of endosomal recycling, Rab11 was shown to regulate the secretion of exosomes in K562 cells [19, 20] and Rab35 regulates exosomes release in oligodendroglial cells by controlling the docking/tethering of vesicles to the plasma membrane [21]. Additionally, our group demonstrated that CA-IKKβ reduces the expression of Rab7 and induces the phosphorylation of SNAP23 at Ser95, which further promotes small EV (sEV) secretion [22]. SNARE proteins form complexes that can mediate the fusion of adjacent plasma membranes as well as that of MVBs with the cell membrane [23]. In K562 cells, VAMP7, a constituent of the SNARE complex, is required for the secretion of exosomes into the extracellular space [24]. In several other cell types, Ca^2+^ can regulate the secretion of exosomes, which may be achieved by activating the SNARE complex [25]. Other SNARE complexes may also be involved in exosome secretion; however, these are not listed here [26, 27].

### Microvesicle biogenesis and secretion

Microvesicles have diameters that range from 50 nm to 1 mm [28]. The plasma membrane undergoes several molecular rearrangements at the sites of microvesicle biogenesis, including changes in Ca^2+^ levels and lipid and protein composition, leading to membrane budding [29, 30]. Changes in Ca^2+^ concentrations lead to the recruitment and activation of calcium-dependent enzymes, which can result in the asymmetric rearrangement of membrane phospholipids and changes in the lipid composition of the plasma membrane [31, 32]. A recent study has also shown that in the resting state, Ca^2+^ mobilization and calpain activation can lead to higher vesiculation levels in malignant (MCF-7) cells than in non-malignant (hCMEC-D3) cells, further confirming that calcium levels play a role in microvesicle formation [33]. Moreover, microvesicles may also arise from cholesterol-rich lipid rafts [34]. Among the proteins involved in microvesicle biogenesis, members of the small GTPase family, such as RhoA, participate in a Rho GTPase-dependent signaling pathway, which triggers the activation of Rho kinase and Lim kinase, finally leading to cofilin phosphorylation and, subsequently, enhanced microvesicle production [35]. Surprisingly, RhoC, similar to RhoA, is also a member of the small GTPase family, cannot induce microvesicle formation in cells, indicating that the signaling mechanism leading to microvesicle biogenesis is highly specific [36]. The release of microvesicles requires them to split from the plasma membrane, which is promoted by the reorganization of the actin–myosin cytoskeleton in a process that may also involve small GTP-binding proteins [37]. Muralidharan-Chari et al. found that GTP/GDP cycling on ARF6 regulates an actomyosin-based membrane abscission mechanism in tumor cells to promote microvesicle release [38]. Similarly, ARF1 affects myosin light-chain (MLC) phosphorylation through modulating RhoA and RhoC activity, which, in turn, promotes the release of microvesicles [39]. Additionally, a recent study reported that lipotoxicity-induced EV release is mediated by the DR5 proapoptotic signaling cascade (CHOP → DR5 → caspase-8 → caspase-3), leading to ROCK1 activation [40].

## The role of PD-L1 in tumor immune escape

### PD-L1 promotes tumor cell immune escape

PD-L1 and PD-L2 (B7DC and CD273) are both ligands for PD-1, an immune checkpoint receptor expressed on the surface of T cells [41, 42]. PD-L1 is mainly expressed on human tumor-associated antigen-presenting cells including tumor environmental dendritic cells (DCs) [43], monocyte-derived myeloid DCs [44], macrophages [43], neutrophils [45], fibroblasts [46], mast cells [47], and other non-tumor cells such as vascular endotheliocytes, keratinocytes, pancreatic islet cells, astrocytes, and corneal epithelial cells [48]. PD-L2 is found on macrophages [49] and DCs [50], among other cells. Importantly, PD-L1 and PD-L2 are co-expressed in a variety of tumor cells. To date, however, evidence to show that blocking PD-L2 or PD-L1 and PD-L2 simultaneously has greater therapeutic efficacy than blocking PD-L1 alone is lacking. As PD-L1 is the main immune checkpoint ligand for PD-1 on T cells in the tumor immune microenvironment [48], we will concentrate more on PD-1/PD-L1-related research, and how this axis mediates tumor immune escape. It is known that PD-L1 on tumor cells interact with PD-1 on T cells, resulting in T-cell dysfunction. Under normal conditions, antigen-activated T lymphocytes can specifically recognize tumor cells and directly kill them, while the combination of PD-L1 and PD-1 may induce T-cell apoptosis, anergy, exhaustion [51–53], and the expression of interleukin 10 (IL-10), a negative regulator of cellular immune responses [54]. However, little is known about the mechanism underlying how PD-1 mediates T-cell dysfunction. T-cell activation requires two types of signals, namely, a T-cell receptor (TCR) signal and a signal from a costimulatory factor, such as CD28. Yokosuka and colleagues showed that PD-1 and TCR can form microclusters, which can reduce the phosphorylation of signaling molecules downstream of TCR by recruiting Src homology 2 domain-containing tyrosine phosphatase 2 (SHP2), resulting in the weakening of T-cell activation [55]. However, Hui et al. found that the combination can lead to the phosphorylation of two tyrosine residues (Y224 and Y248) in the PD-1 cytosolic domain by the lymphocyte-specific protein tyrosine kinase Lck and the subsequent recruitment of SHP2 to dephosphorylate PD-1 and CD28, thereby inactivating CD28 and suppressing T-cell function [3].

### Regulation of PD-L1 expression on tumor cells

**Fig. 1:**
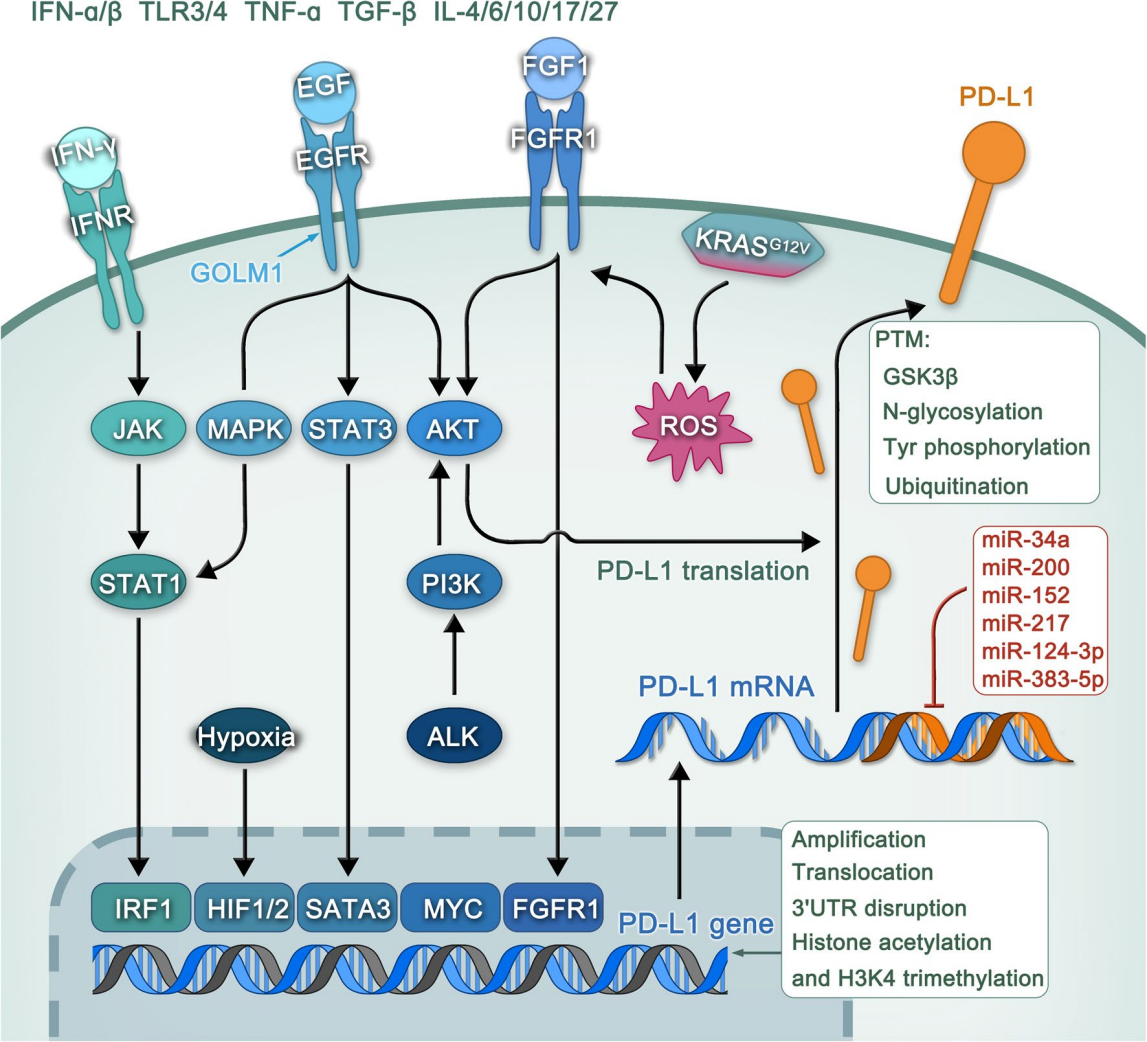
Regulation of PD-L1 expression on the surface of tumor cells. Many factors affect the expression of PD-L1 on tumor cell surface, including genomic alterations and epigenetic, transcriptional, post-transcriptional, and post-translational regulatory mechanisms

Numerous factors influence the expression of the immunosuppressive ligand PD-L1 in the tumor microenvironment, including genomic alterations and epigenetic, transcriptional, post-transcriptional, and post-translational regulatory mechanisms (Table 1 and Fig. 1).

**Table 1:** Regulation of PD-L1 expression on the surface of tumor cells

Stage of regulation	Regulatory mechanism	PD-L1 level	References
Genomic alternations	PD-L1 amplification and translocation in the genome	Up	[56–59]
Genomic alternations	Deletion of the 3'UTR of PD-L1	Up	[60]
Epigenetic regulations	Histone acetylation or methylation of H3K3me3	Up	[61, 62]
Transcriptional level	Upregulation of inflammatory cytokines (e.g., IFN- α/β, IFN-γ, TLR3/4, TNFα, TGFβ and IL-4/6/10/17/27)	Up	[63–74]
Transcriptional level	Aberrant oncogenic signaling pathways up regulate the expression of PD-L1(e.g., MYC, RAS, HIF1/2α, ALK, STAT3, EGFR, PI3K, MAPK)	Up	[75–85]
Post-transcriptional regulation	MiRNAs, including miR-34a, miR-200, miR-152, miR-217, miR-124-3p, and miR-383-5p, can downregulate the expression of PD-L1	Down	[86–93]
Post-translational modification	Interaction between GSK3B and non-glycosylated PD-L1	Down	[94]
Post-translational modification	B3GNT3 promotes the N-glycosylation of PD-L1	Up	[95]
Post-translational modification	Tyr phosphorylation on PD-L1 through the IL-6/JAK1 pathway is necessary for the combination of PD-L1 and the N-glycosyltransferase STT3A to upregulate PD-L1 expression	Up	[96]
Post-translational modification	CSN5 and the deubiquitinase USP22 inhibit PD-L1 ubiquitination and degradation	Up	[97, 98]

#### Genomic alternations and epigenetic regulation

Three main genomic alternations have been associated with increased PD-L1 expression, namely, amplification, translocation, and disruption of its 3'UTR region. *PD-L1* is located on Chromosome 9p24.1, and when this genomic region is amplified, the expression of PD-L1 is significantly increased, as evidenced by poor prognosis and short progression-free survival for patients with Hodgkin's lymphoma, small cell lung cancer (SCLC), non-small cell lung cancer (NSCLC), and other malignant tumors [56–58]. In primary mediastinal large B-cell lymphoma, the PD-L1 locus was specifically rearranged, resulting in the increased expression of PD-L1 [59], and disruption of its 3'UTR region such as delete 3′UTR of *PD-L1* through genome editing can increase its protein expression in many tumors [60]. The expression of PD-L1 on the surface of tumor cells can also be upregulated through epigenetic mechanisms such as histone acetylation and H3K4 trimethylation [61, 62].

#### Transcriptional regulation

##### Inflammatory cytokines

Many inflammatory cytokines are involved in coordinating anti-tumor immunity. These inflammatory cytokines and related inflammatory pathways can also increase the expression of PD-L1 on tumor cells, thereby inhibiting tumor immunity [99]. The interferon family includes two major cytokine-related classes, i.e., type I interferons (IFN-α, IFN-β, and IFN-ω) and type II interferons (IFN-γ), among IFN-γ is a proinflammatory cytokine produced by activated T cells and natural killer (NK) cells, is known to exert effective antiviral and growth-inhibitory effects [100]. Several studies have shown that IFN-γ can induce PD-L1 expression through the IFN-γ/JAK/STAT1 signaling pathway, thereby promoting the immune escape of cancer cells [101, 102]. In addition to IFN-γ, several other inflammatory cytokines also enhance PD-L1 expression on cancer cells or tumor-associated stromal cells, such as IFN-α/β [66], Toll-like receptors3/4 [63, 73], TNF-α [103], TGF-β [104], and IL-4/6/10/17/27 [64, 65, 71, 72, 105]. Interestingly, the detection of the expression of inflammatory cytokines (e.g., IFN-γ, TNF-α, and several ILs) is a predictor of immune checkpoint therapy outcome for advanced NSCLC, while high expression of inflammatory cytokines is positively correlated with anti-PD-1 therapeutic effectiveness [106]. Nevertheless, more data is required to confirm (1) that there is indeed a correlation between inflammatory cytokines and PD-L1 expression in the tumor microenvironment; (2) the specific mechanism underlying a potential correlation; and (3), the effect of inflammatory cytokines on PD-L1 expression in vivo.

##### Oncogenic signaling pathways

In addition to promoting tumor progression in the inherent way of tumor cells, oncogenic signaling pathways can also promote tumor growth by up regulating the expression of PD-L1, thus potentially promoting immune escape. Elucidating how oncogenic signals drive PD-L1 expression can help determine the associated mechanism and provide a therapeutic basis for combining the inhibition of these oncogenic signaling pathways with immune checkpoint therapies for cancer treatment [48]. MYC is one of the most common contributors to tumorigenesis, and its expression is estimated to be elevated or dysregulated in up to 70% of human cancers [107]. MYC has been reported to positively regulate PD-L1 expression in a variety of cancers, including esophageal squamous cell carcinoma [80], NSCLC [79], and lymphoma [75], with evidence indicating that MYC directly regulates the expression of PD-L1 at the transcriptional level [108]. One study reported that RAS also significantly boosted the expression of PD-L1 through a redox-mediated mechanism that RAS activation promoted reactive oxygen species (ROS) production and induced FGFR1 expression, leading to a significant up regulation of PD-L1 expression [76]. Interestingly, Coelho and colleagues demonstrated that RAS can also upregulate PD-L1 expression by increasing PD-L1 mRNA stability via the modulation of the AU-rich element-binding protein tristetraprolin (TTP) [109]. In addition to MYC and RAS, PD-L1 expression can also be upregulated by HIF-1α/2α [81, 85, 110], anaplastic lymphoma kinase (ALK) [82], epidermal growth factor receptor (EGFR) [84], phosphatidylinositol 3-kinase (PI3K) [78], and mitogen-activated protein kinase (MAPK) [83] when they are mutated or overexpressed. Remarkably, many inhibitors that target these oncogenic signaling pathways have been approved by the United States Food and Drug Administration (FDA). These findings highlight the feasibility of combining the inhibition of these oncogenes with immune checkpoint therapy to obtain better treatment effects.

#### Post-transcriptional regulation

MiRNA is a non-coding single-stranded RNA molecule, with about 22–24 nucleotides encoded by endogenous genes, which can regulate post-transcriptional gene expression in animals and plants and play a significant role in intracellular homeostasis and disease [111]. Studies have shown that miRNAs can regulate the expression of PD-L1 either by directly interacting with PD-L1 mRNA or affecting the expression of PD-L1 regulators [48]. Cortez et al. showed that p53 can downregulate the expression of PD-L1 in NSCLC cell lines, an effect that is mediated by the direct binding of miR-34 with the 3′UTR of PD-L1 [88]. Similarly, a recent report demonstrated that miR-34a negatively modulates PD-L1 expression, thereby suppressing the proliferation, metastasis, and invasion of gastric tumor cells [93].Moreover, miR-200 in NSCLC [87]and breast cancer [90], miR-152 in gastric cancer [92], miR-217 in laryngeal cancer [89], miR-124-3p in colorectal cancer [91], and miR-383-5p in breast cancer [86] are thought to play a role in the inhibition of PD-L1 expression.

#### Post-translational modifications

Protein post-translational modification is to increase the functional diversity of proteome through the covalent addition of functional groups or proteins, the proteolytic cleavage of regulatory subunits or the degradation of the whole protein, including phosphorylation, glycosylation, ubiquitination, nitrosylation and methylation, which plays a key role in regulating protein stability, translocation and protein–protein interaction. The post-translational modification of PD-L1 is considered to be an important mechanism of its tumor immunosuppression [2]. Studies have shown that the interaction of glycogen synthase kinase 3 β (GSK3 β) with PD-L1 can induce phosphorylation-dependent proteasome degradation of PD-L1 [94]. Furthermore, EGF can upregulate the expression of β-1,3-galactosyl-O-glycosyl-glycoprotein (B3GNT3) in triple-negative breast cancer cells, thereby promoting the N-glycosylation of PD-L1 and contributing to its interacting with PD-1, finally leading to T cells dysfunction [95]. Chan et al. reported that JAK1 can bind with PD-L1 in the endoplasmic reticulum and indicated PD-L1 Tyr phosphorylation through the IL-6/JAK1 pathway is necessary for the combination of PD-L1 and the N-glycosyltransferase STT3A to upregulate PD-L1 expression [96]. Moreover, COP9 signalosome 5 (CSN5), induced by NF-κB p65, as well as the deubiquitinase USP22, inhibit PD-L1 ubiquitination and degradation, whereas their depletion inhibits tumorigenesis and promotes T-cell cytotoxicity [97, 98].

## The role of EV PD-L1 in tumor immune microenvironment

### PD-L1 loading on EVs

Nucleic acids (including DNA, RNA [mRNA, miRNA, lncRNA]), proteins (including MHC-I, MHC-II, PMEL, TCR, and FasL), and lipids (phosphatidylserine, cholesterol, ceramide) can be delivered to receptor cells as EV contents [4, 28]. The composition of EVs is largely dependent on the cell type and can also be affected by different cellular conditions, including cytoplasmic content [10, 112]. Here, we mainly focus on how PD-L1 is attached to EVs. A recent study identified differences in PD-L1 levels among different cancer cell lines, which could not be explained by the speed of protein translation or protein degradation levels, and the authors speculated that PD-L1 could be secreted from cells in EVs, either in the form of more EVs or as single vesicles carrying more PD-L1 [9]. This suggests that the process involved in loading PD-L1 on EVs may be related to the EV biogenesis process. When the ESCRT-related protein ALG-2 interacting protein X (ALIX) is deleted, the level of PD-L1 on exosomes decreases, and that on cell surface increases, possibly because ALIX is required for the transfer of PD-L1 from the endosomal limiting membrane into MVBs [113]. Similarly, the ESCRT subunit HRS can mediate the identification and sorting of exosome contents, and its knockdown can lead to the decrease of exosomal PD-L1(Exo-PD-L1) level and an increase in that of cellular PD-L1 [8]. In the prostate cancer cell line PC3, when the Rab27a gene, which is related to exosome secretion, and the neutral sphingomyelinase 2 (nSMase2) gene, which promotes the budding of intravesicular vesicles, are knocked out, the levels of PD-L1 and the exosomal marker CD63 are significantly decreased. These data show that Rab27a and nSMase2 play a significant role in the production and secretion of PD-L1-containing exosomes [9]. Many other mechanisms involved in how PD-L1 is loaded onto EVs are currently under investigation, and targeting these mechanisms in combination with anti-PD-L1/ PD-1 therapy has potential as an effective treatment for PD-L1-related cancers.

### The regulation of PD-L1 expression on EVs

The expression of PD-L1 can be influenced by IFN-γ, which is involved in tumor immune escape. Chen et al. found that Exo-PD-L1 has the same membrane topology as PD-L1 on the surface of tumor cells, and the amount of Exo-PD-L1 secreted by tumor cells increased significantly following IFN-γ treatment [8]. Similarly, Ricklefs and colleagues reported that under IFN-γ stimulation, EVs with low PD-L1 expression can inhibit T-cell activation, implying that IFN-γ can also promote PD-L1 expression on EVs [114]. Recently, Chatterjee et al. showed that TGF-β increase the expression of PD-L1 on the exosomes secreted by breast cancer cells in a dose-dependent manner, while blocking exosome release and inhibiting the expression of TGF-β reduced the tumor burden and enhanced T cell toxicity [115]. Also, under TGF-β stimulation, the numbers of PD-L1-containing EVs produced by fibroblasts are increased [116]. Mitochondrial Lon, which functions as a chaperone and DNA-binding protein, plays a role in protein quality control and stress responses. Lon can regulate the metabolism of mitochondrial DNA (mtDNA) and the production of mitochondrial ROS [117]. When Lon is overexpressed, oxidized mtDNA is released into the cytoplasm, IFN production is induced through the cGAS-STING-TBK1 pathway, and the expression of PD-L1 and indoleamine2,3-dioxygenase1(IDO-1) is upregulated, finally leading to the inhibition of T cells activation. Surprisingly, Lon upregulation also induces the secretion of EVs carrying mtDNA and PD-L1 [118]. Radium-223 (Ra-223) was the first bone-homing radiopharmaceutical developed that improved median overall survival (OS) in metastatic prostate cancer patients [119]. The latest findings show that several immune-related factors are enriched in EVs derived from mice treated with Ra-223, including PD-L1, and that ICB/Ra-223 combination therapy can improve the curative effect of anti-tumor therapy [120]. 5-Fluorouracil (5-FU), another key drug for advanced gastric cancer chemotherapy, has also been shown to dose- and time-dependently augment Exo-PD-L1 expression [121]. Recent studies also found that microvesicles from breast cancer cells exposed to radiation carry cargos containing different immunomodulatory proteins, including PD-L1, that inhibit T-cell function and promote tumor growth [122]. Many other cytokines, proteins, and drugs can also affect the expression of PD-L1 on EVs. These merit further investigation because the inhibitors of these factors or the combination of some of these drugs have the potential to improve the curative effect of tumor therapy.

### EV PD-L1 is involved in inducing immune escape in different types of tumors

**Fig. 2:**
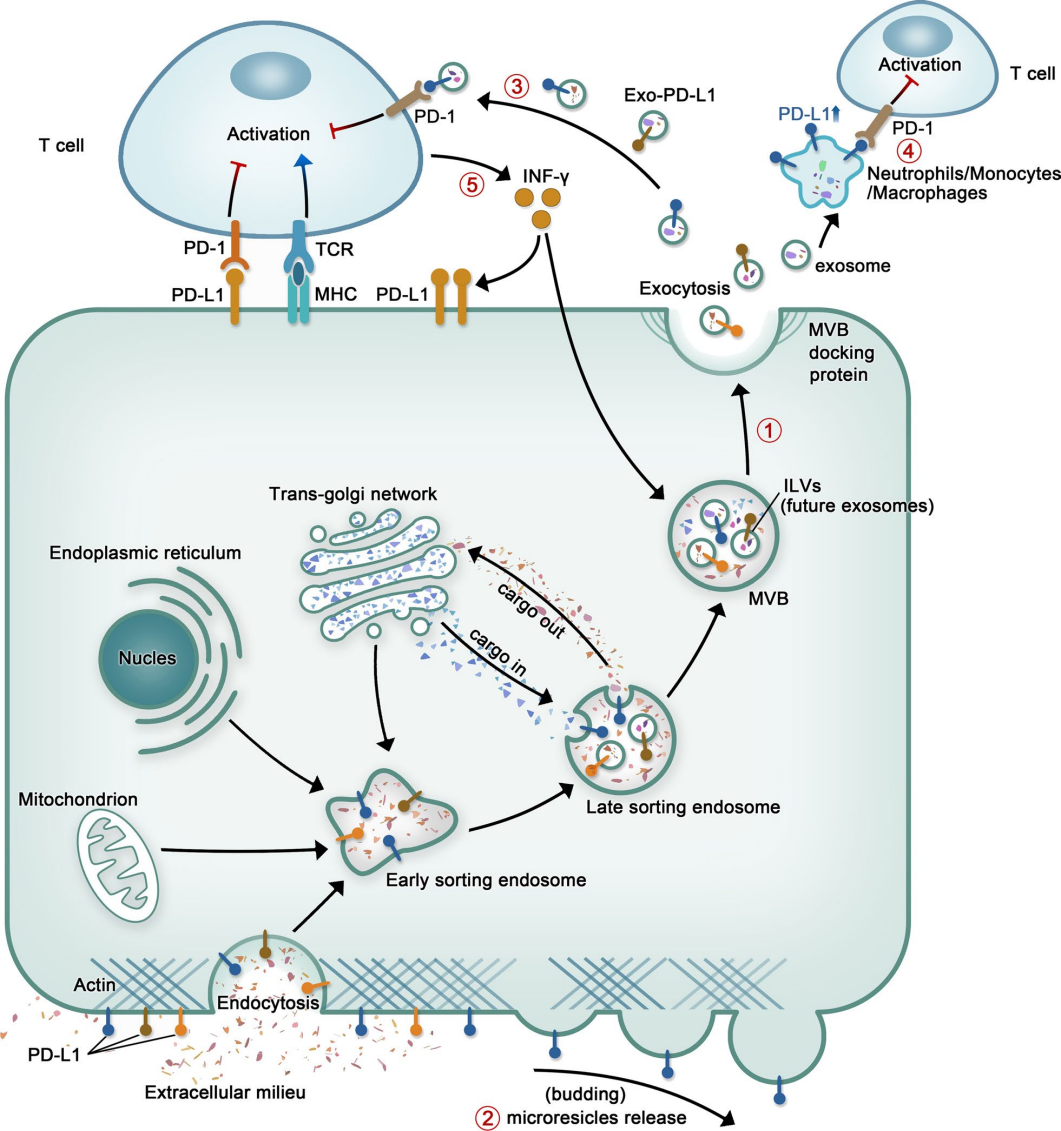
Abbreviated drawing of the formation process of EV-PD-L1 and its direct and indirect inhibitory effects against T cells. ① The process of Exo-PD-L1 production. ②Microvesicles produced by budding can also carry PD-L1. ③ PD-L1 present on the surface of exosomes secreted by tumor cells directly binds to PD-1 on T cells, inducing an immune checkpoint response that inhibits the activation of T cells and disrupts their function, thus inhibiting antitumor immunity. ④ Exosomes released by tumor cells can mediate the increase of PD-L1 expression on the surface of macrophages, neutrophils or monocytes, and then combine with PD-1 on the surface of T cells to inhibit T cells. ⑤ IFN-γ secreted by T cells can promote the expression of PD-L1 on the surface of tumor cells and exosomes

Because immune escape is a major driver of tumor progression, PD-1 and PD-L1, both immune checkpoint-associated proteins, have become the subject of intense investigation. Indeed, immune checkpoint suppressors, mainly those targeting PD-1 and PD-L1, have shown unprecedented prospects and impressive efficacy in the treatment of various human cancers. Nevertheless, the response of a considerable number of cancer patients to this treatment is still poor. Chen et al. revealed that PD-L1 present on EVs displays the same extracellular domain topology as its cell-surface counterpart [8]. As EV PD-L1 may exert functions similar to those of tumor cell surface protein PD-L1 upon PD-1 binding (Fig. 2), EV PD-L1 and their role in tumor immunity have been widely studied over recent years. Several studies have recently reported that PD‐L1 is also detected on EVs in many cancer types, such as prostate cancer [9], melanoma [8], breast cancer [123], head and neck cancer [124], pancreatic cancer [125], glioblastoma [114], gastric cancer [126], and NSCLC [127] (Table 2).

**Table 2:** The effects of extracellular vesicle PD-L1 on tumor cells

Type of tumor	Target cell	Effect	References
*Direct effects*			
Prostate cancer	CD4^+^/CD8^+^T cells	Inhibition of T-cell activation and activity; the percentage of the depletion marker Tim3 increased, while that of the activation marker granzyme B percentage decreased	[9]
Melanoma	CD8^+^T cells	Inhibited the proliferation, cytokine production and cytotoxicity of CD8^+^T cells	[8]
Breast cancer	T cells	Inhibited the indicators of T-cell activation, such as NF-κB activation, as well as PHA-induced interleukin 2 (IL-2) secretion	[123]
Head and neck cancer	CD8^+^T cells	Inhibited the expression of CD69 (a marker of T-cell activation)	[124]
Pancreatic cancer		Exo-PD-L1 expression negatively correlated with postoperative survival time in patients with pancreatic ductal adenocarcinoma	[125]
Glioblastoma	CD4^+^/CD8^+^T cells	The expression of CD69 and CD25 and proliferative ability in CD4^+^ and CD8^+^ T cells decreased	[114]
Gastric cancer	CD4^+^/CD8^+^T cells	Inhibition of T-cell proliferation and negative correlation with granzyme B	[126]
Non-small cell lung cancer	Jurkat cells/CD8^+^T cells	Decreased the production of INF-γ and induced apoptosis	[127]
*Indirect effects*			
Glioblastoma	Monocytes	Extracellular vesicles of gastric cancer cells induce PD-L1 expression on neutrophils to inhibit T-cell-mediated immunity	[133]
Gastric cancer	Neutrophils	EVs of gastric cancer cells induce PD-L1 expression on neutrophils to inhibit T cell immunity	[134]
Chronic lymphocytic leukemia	Monocytes	CLL-derived exosomes increased PD-L1 expression; increased CCL2, CCL4, and IL-6 secretion from monocytes	[138]
Liver Cancer	Macrophages	Hepatoma cells release mir-23a-3p-containing exosomes and upregulate the expression of PD-L1 in macrophages, thereby reducing the ratio of CD8^+^ T cells and promoting T-cell apoptosis	[139]
Non-small cell lung cancer	Macrophages	Exosomes derived from non-small cell lung cancer cells promote the expression of PD-L1 on the surface of macrophages, and then inhibit tumor immunity	[137]

#### Prostate cancer

Most immune checkpoint inhibitors targeting PD-L1 have no effect on prostate cancer patients. Until recently, it was believed that the low PD-L1 expression in prostate cancer cell lines and tissue samples tissues could explain this phenomenon [128]. However, it has since been suggested that prostate cancer cells may secrete PD-L1-carrying EVs, leading to the association of anti-PD-L1 drugs to PD-L1 on the EVs, thereby allowing immune escape to occur. As PD-L1 undergoes endocytosis from the cell surface, the authors premised that PD-L1 was discretely released from in exosomes. Relative to other vesicles, exosomes were enriched by sucrose density gradient centrifugation, and PD-L1 and HRS were found to colocalize with the exosomal marker CD63 in the same sucrose fraction. As previously mentioned, when *Rab27a* and *nSMase2*, genes that are related to exosome biogenesis, are knocked out, the PD-L1 and CD63 levels are significantly decreased [9]. These evidences suggested that PD-L1 could be transported in exosomes. To then elucidate the role of this special form of PD-L1 in the tumor microenvironment, the authors injected wild-type (WT), *Rab27a null*, and *PD-L1 null* TRAMP-C2 cells into the flanks of C57BL6/J syngeneic mice, and, after excluding the key factors of exosomes production, found that Exo-PD-L1 promoted tumor progression. Meanwhile, PD-L1 deletion significantly increased the number, effector function (GzmB), and proliferation (Ki67) of CD8^+^ T cells, and also reduced CD8^+^ T cell exhaustion (Tim-3) in the TRAMP-C2 cancer cells-administered murine draining lymph nodes. Interestingly, when PD-L1-knockout TRAMP-C2 cells were introduced into one side of a mouse, and WT TRAMP-C2 cells into the bilateral side after a few days, WT TRAMP-C2 cells did not develop into tumors in the mouse, indicating that to the presence of PD-L1-deficient tumor cells could generate a strong memory, even to tumor cells that release Exo-PD-L1 [9].

#### Melanoma

PD-L1 was also evident in EVs produced by murine metastatic melanoma B16-F10 cells. The correlation between exosomes and PD-L1 was further confirmed by iodixanol density gradient centrifugation. Because of the low PD-L1 expression in microvesicles, this study focused on Exo-PD-L1. PD-L1 levels in exosomes of metastatic melanoma cells was remarkably elevated, compared to the primary melanoma cells, indicating that the Exo-PD-L1 was strongly correlated with the malignant degree of the tumor [8]. Subsequently, to evaluate the function of Exo-PD-L1 in vivo, the authors generated a murine model of melanoma in C57BL/6 mice utilizing PD-L1-depleted B16-F10 cells. They found that the exosome administration from parental B16-F10 cells could significantly promote tumor growth and reduce quantity of tumor-infiltrating CD8^+^ T lymphocytes (TIL) [8]. Collectively, these data suggest that PD-L1 suppresses antitumor immunity. Recently, Liu et al. demonstrated that the disruption of the glutamic acid–cystine metabolic balance can lead to elevated PD-L1 levels in melanoma via the transcription factors IRF4 and EGR1, promote PD-L1-containing exosomes release, induce M2 macrophage polarization, and reduce PD-1/PD-L1 inhibitor efficacy [129]. These data indicate that glutamic acid–cystine metabolic balance is crucial for immunotherapy, and that the targeted suppression of Exo-PD-L1 release may represent an important means of improving the therapeutic activity of PD-1/PD-L1 suppression.

#### Breast cancer

Yang et al. demonstrated that PD-L1 and CD63 colocalized in MVBs originating from the human breast cancer cell line MDA-MB-231 and human breast cancer tissues via immunofluorescence staining and immunohistochemistry, respectively, thereby confirming that breast cancer cells can also secrete PD-L1-carrying exosomes [123]. Additionally, Exo-PD-L1 dose-dependently inhibited the expression of markers of T-cell activation, such as CD3/CD28-driven ERK phosphorylation and NF-κB activation, as well as PHA-induced IL-2 secretion. The authors further showed that Exo-PD-L1 could interact with PD-1 and suppress T-cell cytotoxicity, thereby promoting tumor growth in vivo [123]. Interestingly, in addition to the role played by breast cancer cell-originating Exo-PD-L1 in tumorigenesis and tumor development, Sun et al. suggested that the Exo-PD-L1 derived from bone marrow significantly increased the lung metastasis of cells from the murine breast cancer line 4T1 by damaging the antitumor CD8^+^ T cell responses at metastatic sites [130].In addition, as the most malignant type of breast cancer, the triple-negative breast cancer cell derived microparticles can also load PD-L1, especially in patients receiving chemoradiotherapy. Microparticles PD-L1 can negatively regulate CD8^+^T cells and polarizing macrophages to M2, resulting in an immunosuppressive microenvironment that promotes tumor progression [131].

#### Glioblastoma

Ricklefs et al. reported finding PD-L1 in glioblastoma-derived EVs, which significantly downregulated levels of early and late activation markers CD69 and CD25 on T cells, respectively, and also reduced the proliferative ability of T cells. Interestingly, further studies revealed that the low PD-L1 levels in plasmoblastoma was upregulated following IFN-γ stimulation, which elevates PD-L1 levels on the EVs, thus inhibiting T-cell activation [114]. Exosomes have also been known to indirectly regulate the immune system via induction of PD-L1 production in a secondary cell type [132]. For example, glioblastoma stem cell-derived exosomes upregulate the expression of PD-L1 in human monocytes, which may correlate with STAT3 phosphorylation [133].

#### Gastric cancer

A multivariate analysis demonstrated that Exo-PD-L1 present in peripheral blood was related to high immunosuppressive activity and poor prognosis in gastric cancer (GC) patients. Furthermore, Exo-PD-L1 was found to exert a more enhanced immunosuppressive response, compared to soluble PD-L1, possibly because exosomal MHC-I promotes Exo-PD-L1-driven T-cell dysfunction [126]. Similar to that seen in glioblastoma, GC-derived EVs transport high-mobility group box-1 (HMGB1) activates signal transducer and activator of transcription 3 (STAT3) and elevates the expression of PD-L1 in neutrophils, thereby inhibiting T-cell immunity [134]. Strategies that interfere with the EV-related HMGB1/STAT3/PD-L1 network have potential as treatments for GC. 5-FU is the main chemotherapeutic agent currently used for the treatment of advanced GC [135], however, recent studies have found that 5-FU promotes a rise in Exo-PD-L1 via the miR-940/Cbl-b/STAT5A network, leading to immunosuppression in patients with late-stage disease [121].

#### Head and neck cancer

PD-L1 was detected in exosomes from patients with head and neck squamous cell carcinoma (HNSCC) by confocal microscopy and flow cytometry, and this form of PD-L1 was associated with disease activity, UICC staging, and lymph node status. In contrast, PD-L1 expressions were not associated with any clinicopathological parameter. Unlike PD-L1^low^ exosomes, exposure to PD-L1^high^ exosomes inhibited CD69 levels, which interferes with the activation of effector T cells [124].

#### Pancreatic cancer

Lux et al. found that Exo-PD-L1 levels were inversely proportional to postsurgical survival duration in patients with pancreatic ductal adenocarcinoma; however, whether pancreatic cancer cells evade the immune response via Exo-PD-L1 requires further investigation [125].

#### NSCLC

PD-L1 was found to be present in extracted plasma exosomes of NSCLC patients in vitro*, *in vivo, and preclinical models. Moreover, Exo-PD-L1 has been reported to inhibit the synthesis of IL-2 and IFN-γ by CD8^+^ T cells, and dose-dependently reduce the overall quantity of CD8^+^ T cells, indicating that Exo-PD-L1 promotes the apoptosis of CD8^+^ T cells and tumor progression via PD-1/PD-L1 interaction [127, 136].In addition, a recent study has shown that exosomes derived from NSCLC can also promote the expression of PD-L1 on macrophages through a NF-κB dependent and glycolysis dominated metabolic reprogramming mechanism, so as to promote tumor metastasis [137].

Apart from to the endogenous Exo-PD-L1 derived directly from solid tumors, chronic lymphocytic leukemia (CLL)-derived exosomes also regulate PD-L1 levels in monocytes. Seiffert et al. identified that, compared with healthy controls, the serum of CLL patients was richer in exosomes from B cells. Sequencing and analysis of the RNA and proteins in the exosomes, respectively, indicated that exosomes from patients with CLL were rich in non-coding Y RNA hY4 (Y RNA is a highly conserved, short, non-coding RNA related to DNA replication and RNA quality control). They also found that hY4 in exosomes derived from CLL patients can bind to TLR7 on the surface of monocytes, thereby promoting the transcription of various inflammatory factors and PD-L1 in monocytes. These events led to the inhibition of tumor immunity and provided a good microenvironment for cancer cell survival [138]. These findings present a potential new direction for CLL immunotherapy. Similarly, endoplasmic reticulum stress promotes the secretion of exosomal miR-23a-3p and the upregulation of PD-L1 levels in macrophages via the phosphatase and tensin homolog (PTEN)/phosphoinositide-4,5-bisphosphate 3-kinase (PI3K)/protein kinase B (AKT) axis [139].

## Potential application of EV PD-L1 in tumors

### EV PD-L1 as biomarker

Tumor immunotherapy has achieved remarkable results over recent years and many clinical studies have shown that immunotherapy can improve the prognosis and significantly prolong the survival time of tumor patients. At the same time, in the context of the trend for precise treatment associated with modern oncology, predictive biomarker detection before treatment can help match individual patients with the most beneficial treatment scheme and reduce the cost of immunotherapy. Consequently, the identification of biomarkers for tumor immunotherapy has gained increasing research interest. PD-L1 has been evaluated as a biomarker of tumor response to immunotherapy [140–142]. However, analysis of the results of preliminary study related to 45 FDA drug approvals encompassing 15 tumor types and carried out from 2011 to April 2019 indicated that PD-L1 is predictive in only 28.9% of these cases, while the number of cases in which PD-L1 is not predictive is as high as 53.3% [143]. Several reasons were proposed to explain the heterogeneity of PD-L1 predictions. First, the type of tumor tissue detected (fresh *vs*. archived), the type of PD-L1 detection method, and PD-L1 expression cutoffs displayed substantial heterogeneity. Secondly, the expression of PD-L1 is regulated by a variety of molecular mechanisms in the tumor microenvironment, and the ability of PD-L1 to drive immunogenicity varies with tumor type [144]. Thirdly, the expression of PD-L1 has temporal and spatial heterogeneity [145] and may also be influenced by prior treatment [144]. In summary, PD-L1 does not appear to be a clinically useful biomarker, meanwhile, people are also actively exploring the role of EVs as tumor immune markers. Therefore, a better biomarker–-EV PD-L1 is needed to guide the choice of immunotherapy (Table 3).

**Table 3:** EV PD-L1 as a potential biomarker for tumor diagnosis, progression and treatment

Type of tumor	Biomarker type	Effect	References
Metastatic melanoma	Diagnostic biomarker	Exo-PD-L1 levels in patients is higher than those in healthy controls	[8]
NSCLC	Diagnostic biomarker	Exo-PD-L1 levels patients in is higher than those in healthy controls	[136]
HNSCC	Tumor progression biomarker	The RFV of Exo-PD-L1 in patients with high UICC stage was higher than that in patients with low UICC stage	[124]
NSCLC	Tumor progression biomarker	High levels of Exo-PD-L1 were associated with larger tumor size, positive lymph node status, distant metastasis and advanced TNM stage	[136]
Pancreatic cancer	Tumor progression biomarker	The OS of patients with high Exo-PD-L1 levels was markedly lower	[125]
GC	Tumor progression biomarker	The OS of patients with high Exo-PD-L1 levels was markedly lower	[126]
Osteosarcoma	Tumor progression biomarker	The levels of Exo-PD-L1 were positively correlated larger tumor size	[114]
Glioblastoma	Tumor progression biomarker	Exo-PD-L1 is associated with lung metastasis of osteosarcoma	[146]
Melanoma	The marker of the efficacy of ICB	High levels of Exo-PD-L1 are associated with low response to anti-PD-1 therapy	[8, 147]
NSCLC	The marker of the efficacy of ICB	The level of Exo-PD-L1 was lower in patients with effective anti-PD-1 therapy	[147]

EV PD-L1 can be used as a diagnostic marker for various tumors, such as the Exo-PD-L1 levels in metastatic melanoma and NSCLC patients (especially those in advanced stages) were reported to be higher than those in healthy controls [8, 136]. Based on the role of EV PD-L1 as a diagnostic biomarker, some efficient methods for detecting Exo-PD-L1 have gradually emerged. For instance, Fe3O4@TiO2 nanoparticles were designed to enrich and separate exosomes from solution, and displayed a capture rate of 96.5% within 5 min. Subsequently, anti-PD-L1 antibody-modified Au@Ag@MBA SERS tags were utilized to quantify Exo-PD-L1 levels [148]. Additionally, a new PD-L1 aptamer, which not only has good selectivity but also interacts with natural PD-L1 more efficiently than antibodies, was combined with thermophoresis to yield a uniform, low volume, efficient, and sensitive method for the quantitation of Exo-PD-L1 (HOLMES-ExoPD-L1) [149]. Wang et al. used gold-silver (Au@Ag) core–shell nanobipyramids and gold nanorods to produce a unique plasma signal pattern, allowing rapid and highly sensitive exosome detection and accurate identification of Exo-PD-L1 [150].

In patients with HNSCC, the relative fluorescence values (RFVs) for Exo-PD-L1 were higher in patients with active disease than those without evidences of disease, and were also higher in patients with high UICC staging (III and IV) compared with those with low UICC staging (I and II) [124]. Similarly, in NSCLC patients, higher levels of Exo-PD-L1, rather than soluble PD-L1, were reported to be interrelated to larger tumor size, positive lymph node status, distant metastasis, and late TNM staging [136]. These observations indicate that Exo-PD-L1, rather than soluble PD-L1, may be a clinically relevant variable with potential predictive value in HNSCC and NSCLC. In addition, compared with those who have low levels of Exo-PD-L1, the OS of pancreatic cancer and GC patients with high Exo-PD-L1 levels was markedly lower; thus, PD-L1 expression on exosomes can be considered to be a negative prognostic factor for these cancers [125, 126]. Moreover, the amount of PD-L1 DNA contained in serum and plasma EVs obtained from glioblastoma patients was positively correlated with the size of the tumor [114]. Osteosarcoma is a major malignant bone tumor, with approximately 15%–20% of patients exhibiting lung metastasis [151]. Osteosarcoma cells can stimulate lung metastasis by releasing exosomes carrying PD-L1 and N-cadherin, indicating that the test of exosomes carrying PD-L1 and N-cadherin in serum can be used as a predictor of lung metastasis in osteosarcoma patients [146].

In addition to tumor diagnosis and progression, PD-L1 is also the marker of the efficacy of tumor immune checkpoint inhibitors. The levels of Exo-PD-L1 were remarkably higher in melanoma patients who did not respond to anti-PD-1 drugs than in those who did [8]. Cordonnier and colleagues suggested that PD-L1 may also be a predictor of curative effect in melanoma patients, and has the advantages of noninvasive collection and real-time monitoring [152]. Furthermore, Exo-PD-L1 expression levels were significantly lower in melanoma and NSCLC patients responding to anti-PD-1 treatment compared with those in patients with disease progression [147]. In addition to lower levels of Exo-PD-L1, patients who responded to anti-PD-1 therapy also exhibited a higher level of CD28 expression, so the combination of Exo-PD-L1 with serum CD28 may be an effective marker for predicting the response to anti-PD-1 therapy [153].

### The significance of EV PD-L1 in therapy

**Fig. 3:**
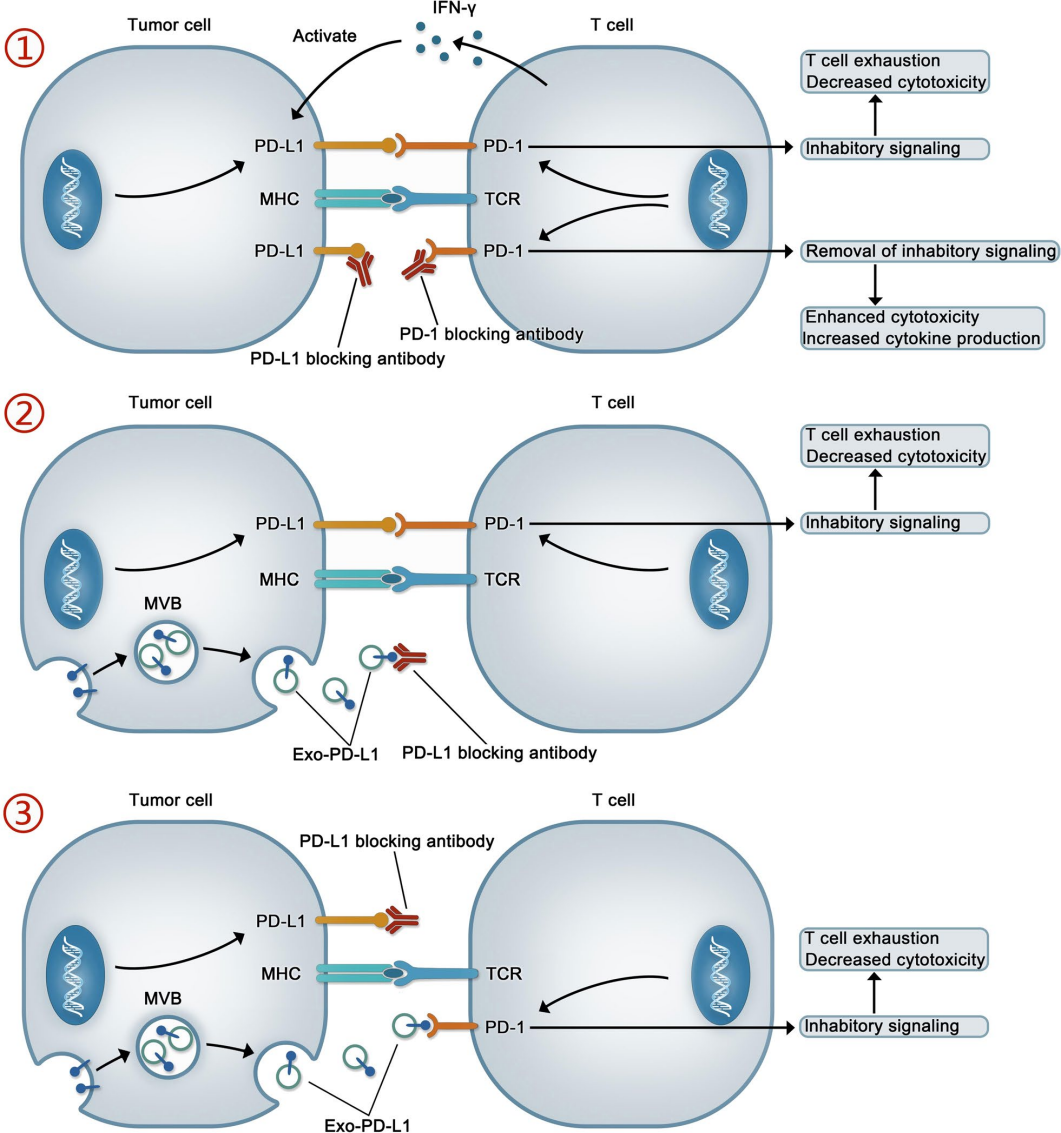
The mechanism of action of anti-PD-1/PD-L1 drugs and the potential mechanism underlying the exosomal PD-L1-mediated resistance to anti-PD-L1 drugs. ① Anti-PD-1/PD-L1 drugs can interact with PD-1/PD-L1, free T cells from the checkpoint block, and restore immune responses. ② and ③ Exo-PD-L1 is thought to contribute to resistance during immunotherapy through two mechanisms. In one, exo-PD-L1 binds to anti-PD-L1 monoclonal antibody (mAb), leading to PD-L1 exposure on the tumor surface (②); in the other, Although anti-PD-L1 mAb can interact with PD-L1 on the surface of tumor, exo-PD-L1 can directly interact with PD-1 on the surface of T cells to inhibit immunity (③)

In recent years, immune checkpoint therapy has been increasingly used as an important treatment for various cancer. Although immune checkpoint therapy improves the prognosis of patients with various types of cancer, only fews of these patients have achieved long-term benefits. Why most patients do not respond to or cannot maintain their response to immune checkpoint therapy is the subject of intense investigation. The mechanisms underlying drug resistance are usually divided into two types—primary and acquired. With primary drug resistance, patients have no initial response to immune checkpoint blockade, while with acquired resistance, patients initially respond to immune checkpoint therapy but later become refractory to treatment [154, 155]. A substantial amount of convincing evidence exists for the occurrence of primary resistance to drugs targeting EV PD-L1, which may underlie the relatively low response rate to anti-PD-L1/PD-1 therapy [8, 126]. Although the specific mechanisms involved in the EV PD-L1-mediated resistance to anti-PD-L1/PD-1 therapy remain largely unclear, we can make a guess about it, as shown in Fig. 3.

EVs, as an essential material information exchange carrier in the tumor microenvironment, can also play a double-edged sword role. It is undeniable that EVs released by DCs and genetically engineered T cells expressing chimeric antigen receptor (CAT) play an emerging role in tumor immunotherapy [156]. However, EV PD-L1 or EV carrying another immune checkpoint such as LGALS9 can play a negative role in tumor immunity [157]. Similarly, EV also carries some monoclonal antibody targets or RNA, leading to failure of treatment, such as EV released from breast cancer cells contains HER2 protein or AFAP1-AS1 (actin filament associated protein 1 antisense RNA 1), which will affect the efficacy of trastuzumab [158, 159]. In addition, exosomes can also promote the formation of tumor cell pre-metastatic niche [160]. Therefore, exosomes may become the target of tumor therapy. One method is to block the release of exosomes, in which GW4869 is the most commonly used exosome inhibitor [161]. Dai et al. developed the assembled nanounits of GW4869 and iron death inducer (Fe3^+^) to reduce the secretion of tumor derived exosomes and weaken the immunosuppressive effect of exo-PD-L1, which induce anti-tumor immune response of melanoma cells and stimulate cytotoxic T lymphocytes and immune memory [162]. Similarly, amiloride, a calcium exchanger inhibitor, is considered to be an inhibitor of exosome release [163, 164]. At the experimental level, Rab27A null TRAMP-C2 cells were generated through CRISPR/cas9-mediated deletion and injected into C57BL6/J mice. No tumor growth was detected in these animals, even after more than 4 months [9]. Similarly, in a murine model of breast cancer, Rab27A knockdown in a 4T1 cell line markedly inhibited tumor progression and enhanced anti-PD-L1 curative effect [123]. Notably, in MC38 cell lines, the combination of exosome removal and anti-PD-L1 therapy prolonged the survival time of mice to a degree similar to that of PD-L1 null cell lines [9]. Another method is to target the released exosomes, such as, a recent study designed and synthesized a new compound called Exoblock, which has the characteristics of high affinity binding to phosphatidylserine, was found to significantly block the immunosuppressive activity of human ovarian cancer and melanoma related exosomes [165].In conclusion, these findings suggest that the elimination of EVs has potential for use as an effective adjunctive therapy for improving the efficiency of anti-tumor therapy. Interestingly, a recent study constructed an engineered MDA-MB-231 cell line, which overexpressed high-affinity mutant human PD-1 protein (havPD-1) and knocked out endogenous PD-L1 and β-2 microglobulin microglobulin. HavPD-1 EVs produced by this cell line reduced the proliferation of PD-L1 overexpressed cancer cells and induced apoptosis [166].

In eukaryotes, cells can respond to external stimuli through autophagy and exosome secretion to maintain cell homeostasis. Autophagy is a lysosome-dependent mechanism for the degradation of cellular components and can be induced by oxidative stress, starvation, or protein aggregation. Amphisomes are intermediate organelles formed through the fusion of autophagosomes and exosomes [167]. It was recently reported that PD-L1 on exosomes can influence tumor autophagy. For example, glioblastoma stem cell-derived, PD-L1-containing exosomes can activate AMPK1/ULK1 signaling cascade-mediated autophagy, thus increasing the resistance of glioblastoma to temozolomide [168]. Importantly, this study may lead to an alternative strategy for the treatment of glioblastoma.

The immunosuppressive effect of EV PD-L1 is well known; however, this type of PD-L1 has also been associated with positive effects. A recent study suggested the inhibitory effect of PD-L1 may promote tissue repair [169] as excessive and persistent proinflammatory activity after trauma can aggravate tissue damage [170, 171]. Exo-PD-L1 can promote the migration of epidermal cells and dermal fibroblasts, markedly accelerating wound contraction and reepithelization in a mouse model of skin excision injury. Additionally, Exo-PD-L1 also inhibited the production of cytokines as well as the number of CD8^+^ T-cells in the spleen and peripheral lymph nodes [169]. In summary, Exo-PD-L1 plays an immunosuppressive role and promotes tissue repair.

## Conclusions

Tumor immune escape plays a significant role in tumor occurrence and development, and can also partly explain the failure of immunotherapy. PD-L1 derived from tumor cells can interact with PD-1 on immune cells, thereby inhibiting the activity of T cells. Meanwhile, EVs can carry nucleic acids, proteins, lipids, and other molecules into the systemic circulation and transport them to all parts of the body, thereby participating in intercellular communication. Many studies involving tumor models have shown that EV PD-L1 plays a significant role in the immune escape of several cancer types. Tumor cells release EV PD-L1, which can interact with PD-1 on the surface of T cells, thereby inhibiting their effector function and reducing the release of the pro-inflammatory cytokines IFN-γ, IL-2, and granzyme-b. There are two main forms of EVs—exosomes and microvesicles—both of which can carry PD-L1. However, due to the complexity of methods associated with the separation and purification of microvesicles, most research to date has focused on Exo-PD-L1. However, with the progress of research methods, this is likely to change in the future. EV PD-L1 can also exert its immunosuppressive activity through indirect mechanisms (Table 2 and Fig. 2). These observations indicate that the mechanisms underlying immune escape are very complex and that PD-L1 released in EVs is merely the tip of the iceberg. There is still a long way to go to achieve effective cancer treatment.

In conclusion, we summarized the immunosuppressive effect of EV PD-L1 in many tumor models as well as its potential role as the marker of early cancer diagnosis, tumor progression, and tumor-targeting immunotherapy. However, many questions remain unanswered, including whether all tumors produce and secrete PD-L1-carrying EVs, whether all EV PD-L1 will lead to immunosuppression, and whether other mechanisms or signaling pathways are also involved in this inhibition. Furthermore, although EV PD-L1 can sequester anti-PD-L1 drugs, thus contributing to drug resistance, the specific mechanism underlying its involvement in the resistance to anti-PD-1/PD-L1 immunotherapy is still unclear. Moreover, to date, EV PD-L1 has not been widely used as the biomarker in clinical practice.

## Data Availability

No data involved.

## Abbreviations

ALIX: ALG-2 interacting protein X
ALK: Anaplastic lymphoma kinase
AFAP1-AS1: Actin filament associated protein 1 antisense RNA 1
ARF6: ADP-ribosylation factor 6
ARF1: ADP- ribosylation factor 1
B3GNT3: β-1,3-Galactosyl-O-glycosyl -glycoprotein
CLL: Chronic lymphocytic leukemia
CSN5: COP9 signalosome 5
DCs: Dendritic cells
EVs: Extracellular vesicles
Exo-PD-L1: Exosomal PD-L1
ESEs: Early-sorting endosomes
EGFR: Epidermal growth factor receptor;
ESCRT: Endosomal sorting complex required for transport
FDA: Food and Drug Administration
GC: Gastric cancer
GTP: Guanosinetriphosphate
GDP: Guanosine diphosphate
GSK3B: Glycogen synthase kinase 3B
HNSCC: Head and neck squamous cell carcinoma
havPD-1: High affinity mutant human PD-1 protein
HMGB1: High-mobility group box-1
HRS: Hepatocyte growth factor-regulated tyrosine kinase substrate
IDO-1: Indoleamine2,3-dioxygenase1
IFN-γ: Interferon-gamma
IL-10: Interleukin 10
ILVs: Intraluminal vesicles
LSEs: Late-sorting endosomes
MAPK: Mitogen-activated protein kinase
MLC: Myosin light-chain
MVBs: Multivesicular bodies
mtDNA: Mitochondrial DNA
NK: Natural killer
nSMase2: Neutral sphingomyelinase 2
NSCLC: Non-small cell lung cancer
OS: Overall survival
PD-L1: Programmed cell death 1 ligand 1
PD-1: Programmed death protein-1
PI3K: Phosphatidylinositol 3-kinase
PTEN: Phosphatase and tensin homolog
RFVs: Relative fluorescence values
Ra-223: Radium-223
ROS: Reactive oxygen species
STAT3: Signal transducer and activator of transcription 3
SHP2: Src homology 2 domain-containing tyrosine phosphatase 2
SCLC: Small cell lung cancer
SNARE: Soluble N-ethylmaleimide-sensitive factor attachment protein receptor
sEV: Small EV
TCR: T-cell receptor
TNF-α: Tumor necrosis factor α
TGF-β: Transforming growth factor β
TTP: Tristetraprolin
VAMP7: Synaptic vesicle-associated membrane protein 7
VPS4: Vacuolar protein sorting 4
WT: Wild-type
5-FU: 5- Fluorouracil
